# A Method for Prediction of Thermophilic Protein Based on Reduced Amino Acids and Mixed Features

**DOI:** 10.3389/fbioe.2020.00285

**Published:** 2020-05-05

**Authors:** Changli Feng, Zhaogui Ma, Deyun Yang, Xin Li, Jun Zhang, Yanjuan Li

**Affiliations:** ^1^College of Information Science and Technology, Taishan University, Tai’an, China; ^2^Department of Rehabilitation, General Hospital of Heilongjiang Province Land Reclamation Bureau, Harbin, China; ^3^Information and Computer Engineering College, Northeast Forestry University, Harbin, China

**Keywords:** thermophilic protein, reduced amino acids, mixed features, machine learning methods, non-thermophilic protein

## Abstract

The thermostability of proteins is a key factor considered during enzyme engineering, and finding a method that can identify thermophilic and non-thermophilic proteins will be helpful for enzyme design. In this study, we established a novel method combining mixed features and machine learning to achieve this recognition task. In this method, an amino acid reduction scheme was adopted to recode the amino acid sequence. Then, the physicochemical characteristics, auto-cross covariance (ACC), and reduced dipeptides were calculated and integrated to form a mixed feature set, which was processed using correlation analysis, feature selection, and principal component analysis (PCA) to remove redundant information. Finally, four machine learning methods and a dataset containing 500 random observations out of 915 thermophilic proteins and 500 random samples out of 793 non-thermophilic proteins were used to train and predict the data. The experimental results showed that 98.2% of thermophilic and non-thermophilic proteins were correctly identified using 10-fold cross-validation. Moreover, our analysis of the final reserved features and removed features yielded information about the crucial, unimportant and insensitive elements, it also provided essential information for enzyme design.

## Introduction

Proteins denature when the environmental temperature increases dramatically (Tang et al., 2017). However, thermophiles can survive in temperatures ranging from 41°C to 122°C (Takai et al., 2008; Fan et al., 2016) and produce enzymes that react well at higher environmental temperatures, such as 120°C (Fan et al., 2016). In enzyme engineering, identifying the functional mechanisms of these proteins will provide insights into the design and optimization of enzymes (Tang et al., 2017).

Protein thermostability has been shown to be related to hydrophobicity (Gromiha et al., 2013), hydrogen bonding (Bleicher et al., 2011), hydrophobic free energy (Gromiha et al., 1999; Saraboji et al., 2005), and residue (Meruelo et al., 2012) and inter-residue contacts (Gromiha, 2001). Moreover, Das and Gerstein (2000) found that salt bridges are essential for maintaining protein thermostability in thermophilic bacteria. The distribution of amino acids in proteins (Fukuchi and Nishikawa, 2001; Zhou et al., 2008) and the presence of dipeptide (Ding et al., 2004; Zhang and Fang, 2006a, b) also affect protein thermostability. In a study by Vieille, the composition of Arg is greater in thermophiles than in mesophiles (Vieille and Zeikus, 2001). Guo also showed that expurgation of water-accessible thermo-labile residues, such as Gln and Met, affects the thermostability of enzymes expressed by thermophiles (Guo et al., 2014). Besides, Chen et al. (2016) found the pseudo amino acid composition had a big effect on the protein identification task, and constructed a web server to give a free way to use their algorithm^1^.

Sequence-based protein identification provides an alternative method for studies of protein thermostability (Zhang and Fang, 2007; Wu et al., 2009; Li and Fang, 2010; Liu et al., 2011, 2019; Zuo et al., 2013; Fu et al., 2018; Wang et al., 2018; Zhang et al., 2018; Cheng et al., 2019; Yu et al., 2019b). Wang et al. (2011) introduced a feature selection method to identify vital features from the pseudo amino acid composition, amino acid composition, physicochemical features, composition transition, and distribution features using a support vector machine (SVM) to detect thermophilic proteins. Additionally, Tang proposed a two-step discrimination method with 94.44% accuracy using 5-fold cross-validation. Lin et al. constructed a dataset containing 915 thermophilic proteins and 793 non-thermophilic proteins, and predicted 93.8% thermophilic proteins and 92.7% non-thermophilic proteins using SVM. The same conclusion was also reached by Nakariyakul et al. (2012), who obtained 93.3% identification accuracy in the same database used by Lin. In another study, Fan et al. (2016) integrated information on the amino acid composition, evolution information, and acid dissociation constant to identify thermophiles by SVM, yielding an overall accuracy of 93.53%. Modarres et al. (2018) proposed a new thermophilic protein database, which contained 14 million protein sequences. In this database, all sequences were categorized according to the thermal stability and protein family property. Not only the sequences but also structures of thermophilic proteins were contained in the database. This online database gave the developers a powerful tool in the thermophilic protein prediction task.

In this study, we integrated 188 physicochemical characteristic features, auto-cross covariance (ACC) information, and dipeptide compositions of reduced amino acids to obtain a mixed feature set. Redundant features were then removed using correlation analysis, and dimensions were reduced using the max-relevance-max-distance (MRMD) method and principal component analysis (PCA). Finally, the SVM and other three machine learning methods were used to identify thermostability.

## Materials and Methods

**FIGURE 1:**
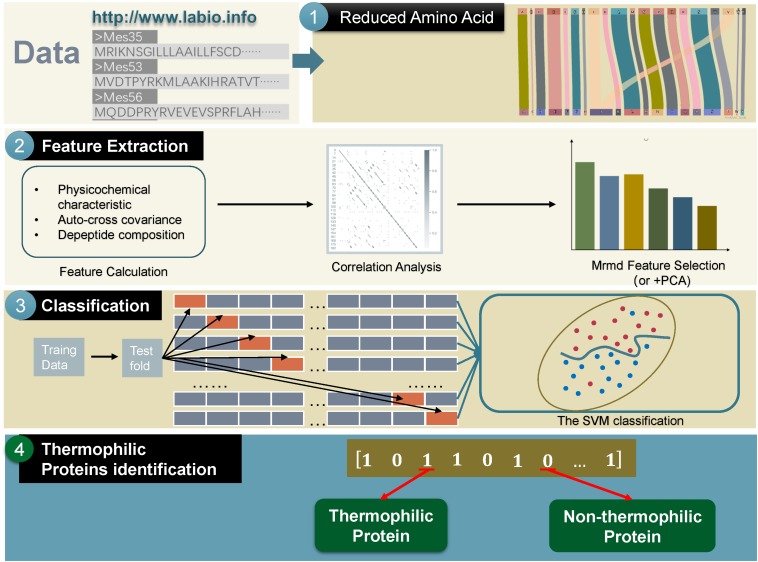
The whole framework of the proposed method in this manuscript.

The main framework of the method used in this study could be divided into the following four parts: (a) transforming thermophilic protein sequences to a reduced amino acid form; (b) extracting useful features; (c) using the SVM to train the extracted features; (d) predicting the test data by machine learning (Yu et al., 2017a, b; Zou et al., 2017a, b; Zhang et al., 2019a). The framework is shown in Figure 1.

### Datasets

We used the dataset constructed by Lin et al. (Lin and Chen, 2011), whose data were chosen from the Universal Protein Resource (UniProt). The temperature of thermophilic proteins in this dataset was set to above 60°C and the temperature of non-thermophilic proteins was set to be less than 30°C. After removing redundancy and homology bias, there were 915 thermophilic and 793 non-thermophilic proteins. These data can be downloaded from http://www.labio.info/index-1therm.html.

### Reduced Amino Acid Composition (RAAC)

In order to improve phylogenetic estimates, it is possible to recode the amino acids in the protein sequence (Susko and Roger, 2007). Furthermore, some reduced amino acid schemes, including the “Dayhoff classes” (AGPST, DENQ, HKR, ILMV, FWY, and C), have attracted attention (Susko and Roger, 2007).

In order to maximize the ratio of the expected number of substitutions within bins under the JTT model, Susko et al. proposed their reduced amino acid alphabet, which contains 30 schemes. In this study, we chose the final scheme as follows: A, C, D, E, F, G, H, IV, K, L, M, N, P, Q, R, S, T, W, Y. Thus, the 20 amino acids were classified into 19 types in the above scheme (Susko and Roger, 2007), in which Ile (I) and Val (V) were viewed as a single type, while every one of other categories had only one amino acid. Under this reduced scheme, we use the webserver of Zuo (Zheng et al., 2019) to calculate the RAAC of the thermophilic and non-thermophilic proteins.

Furthermore, dipeptides of proteins, like AA, A^∗^A (λ_*gap*_ = 1), and A^∗∗^A (λ_*gap*_ = 2), AK, A^∗^K, A^∗∗^K, etc., were also obtained using this webserver (Chen et al., 2016; Yang et al., 2019). The following formula was used to calculate the values of those features:

$$f_{361}^{\lambda }\,\left(j\right)=\frac{y_{361}^{\lambda }\,\left(j\right)}{\sum_{j}y_{361}^{\lambda }\,\left(j\right)}\;\lambda =0,1,2,\cdots ,361,$$

where $y_{361}^{\lambda }\,\left(j\right)$ denotes the number of λ-gap dipeptides of type *j* in a protein sequence.

### Feature Extraction

#### Physicochemical Characteristics

To quantitatively identify proteins, the physicochemical characteristics were obtained using a method (temporarily called 188d), which could extract sequence information and amino acid properties (Song et al., 2014; Xu et al., 2014, 2018; Fu et al., 2019; Liu, 2019; Zhu et al., 2019). The first 20 elements in the results of this method denoted the frequency of the 20 original amino acids (Zhu et al., 2019); the next 24 features reflected the group proportion corresponding to three groups (Qu et al., 2019); the following 120 dimensions were the distributions of three groups in five local positions (Cai et al., 2003); the last 24 features were the numbers of three types of dipeptides.

#### ACC

Auto covariance (AC) and cross-covariance (CC) calledACC, can reflect the relationship between amino acids with certain length features and contains AC and CC (Dong et al., 2009; Liu et al., 2015). The formula of CC transforms a protein sequence to a vector form Liu et al. (2016):

$$P^{\prime }=\left[\varphi_{1},\varphi_{2},\varphi_{3},\cdots ,\varphi N*\left(N-1\right)*lg\right)]{}^{T},$$

where N denotes the number of properties. φ_*i*_ can be calculated as:(Guo et al., 2008)

$$\varphi_{n}=A\,C\,\left(i,l\,g\right)=\frac{1}{N-l\,g}\,\sum_{j=1}^{L-l\,g}\left(S_{i,j}-\bar{S_{i}}\right)\,\left(S_{i,j+l\,g}-\bar{S_{i}}\right),$$

where *i* is a residue, *L*denotes the length of the whole protein sequence, *S*_*i,j*_ represents the *i*-th property of the *j*-th amino acid, and *S*_*i*_ reflects the mean value of the *i*-th property (Qu et al., 2019). In our experiment, the value of *lg* was set to 2.

#### Correlation Analysis

Some pairs in our feature set were found to be highly correlative, indicating that the effects of these two features were similar. Furthermore, this phenomenon denotes redundant and repeated information were present in the feature set. However, without the preprocess of discarding redundant information, machine learning models are associated with a risk of overfitting (Hua et al., 2009; Mwangi et al., 2014; Zeng et al., 2019b).

Thus, a correlation analysis-based redundant information expurgate method was proposed to discard one feature from each of the highly relevant feature pairs. As a prepare step, all feature values need to be normalized to [0,1] using the following equation:

$$x_{i}^{n}=\frac{x_{i}-\bar{x}}{x_{m\,a\,x}-x_{m\,i\,n}},$$

where *x*_*i*_(*i* = 1,2,3,⋯) denotes the *i*-th value in the feature set, $\bar{x}$represents the mean value of the current feature vector, and*x*_*max*_, *x*_*min*_ correspondingly reflect the maximum and minimum values of the feature vector.

Then, Pearson’s correlation was used to evaluate the correlations between any two features. Its value was written as follows:(Thibeault and Srinivasa, 2013; Jin et al., 2019)

$$\rho \,\left(X,Y\right)=\frac{1}{n-1}\,\sum_{i=1}^{n}\left(\frac{X_{i}-\bar{X}}{\sigma \,\left(X\right)}\right)\,\left(\frac{Y_{i}-\bar{Y}}{\sigma \,\left(Y\right)}\right),$$

where *X* and *Y* are two given feature vectors, $\bar{X}$ and $\bar{Y}$ represent the mean value of *X* and *Y*, respectively, and σ(*X*) and σ(*Y*) denote the standard deviations of *X* and *Y*, respectively.

In our experiment, for any feature pair *X* and *Y*, if the value of ρ(*X*,*Y*) was larger than the threshold *T*, then *X* and *Y* were considered a highly correlated feature pair. In the next step, we decided whether to remove one of the features from the feature set while retaining the other in the feature set. Thus, for the first feature pair, a removed feature set *D* and a reserved feature set *R* were created and set as an empty set. Then, one feature was set to belong to *D*, while the other was set to belong to *R* randomly. In the following computation, the rule for assigning features could be expressed as follows: assuming that *X*-*Y* is a highly correlative feature pair,

• If *X*∉*D* and *X*∉*R*: *Y*∉*D* and *Y*∉*R*→*Y* ∈ *D*, *X* ∈ *R*
• If *X*∉*D* and *X*∉*R*: *Y* ∈ *D*→*X* ∈ *R*
• If *X*∉*D* and *X*∉*R*: *Y* ∈ *R*→*X* ∈ *D*
• Elseif *X* ∈ *R* : *Y*∉*D* and *Y*∉*R*→*Y* ∈ *D*
• Elseif *X* ∈ *D* : *Y*∉*D* and *Y*∉*R*→*Y* ∈ *D*

Let $D=\left\{f_{1}^{\prime },f_{2}^{\prime },f_{3}^{\prime },\cdots ,f_{M}^{\prime }\right\}$ denote the final removed feature set. After all *M* features in *D* were removed from the feature set, the correlation between feature pairs was decreased dramatically. The threshold *T* used in our experiment was set as 0.85.

#### MRMD Feature Selection

Dimensionality reduction is a key process in machine learning research and application (Bhola and Singh, 2018). The MRMD method, as presented by Zou et al. (2016), was used to rank features in descending order and reduce the feature number. There were two object functions; the first reflected the relationship between the current feature and the target class, which could be written as follows (Zou et al., 2016):

$$P\,P\,C\,\left(\vec{F_{i}},\vec{C_{i}}\right)$$

$$=\frac{\frac{1}{N-1}\,\sum_{k=1}^{N}\left(f_{i,k}-\bar{f_{i}}\right)\,\left(C_{i,k}-\bar{C_{i}}\right)}{\sqrt{\frac{1}{N-1}\,\sum_{k=1}^{N}{\left(C_{i,k}-\bar{C_{i}}\right)}^{2}}\,\sqrt{\frac{1}{N-1}\,\sum_{k=1}^{N}{\left(f_{i,k}-\bar{f_{i}}\right)}^{2}}},$$

$$m\,a\,x\,M\,R_{i}=\left|P\,P\,C\,\left(\vec{F_{i}},\vec{C_{i}}\right)\right|,$$

where *f*_*i,k*_ and *C*_*i,k*_ represent the *k*-th element in the feature vector *F*_*i*_ and *C*_*i*_, respectively. The other object function was expressed in the following form Zou et al. (2016):

$$E\,D\,\left(\vec{X},\vec{Y}\right)=\sqrt{\sum_{k=1}^{N}{\left(x_{k}-y_{k}\right)}^{2}},$$

$$\max M\,D_{i}=E\,D_{i}=\frac{1}{M-1}\,\sum E\,D\,\left(\vec{F_{i}},\vec{F_{k}}\right).$$

Integrating the above two functions, we obtained the final objective function, which was written as follows:

$$m\,a\,x\,\left(M\,R_{i}+M\,D_{i}\right)$$

Solving this function, when the function reached the maximum ACC value, the iteration was stopped automatically, giving a feature dimension reduced set.

#### PCA

Principal component analysis (Price et al., 2006) is a widely used tool that can transform the features of observation into an uncorrelated feature set (Zeng et al., 2017, 2019a; Xiao et al., 2018; Zhang et al., 2019b). The main steps of PCA are as follows: (1) normalize the feature vector value; (2) calculate the covariance matrix by $\sum =\frac{1}{m}\,X\cdot X^{T}$; (3) use the singular value decomposition method (*U*,*S*,*V^T^*); = *SVD*(Σ); (4) extract the first k singular vectors from U and (5) calculate the i-th eigenvalue λ_*i*_,*i* = 1,2,3,⋯

We used ρ to evaluate the cumulative contribution value of the singular vectors; this value was defined as $\rho =\frac{\sum_{i=1}^{p}\lambda_{i}}{\sum_{i=1}^{m}\lambda_{i}}\geq T{}^{\prime }$, where m denotes the dimension of the transformed features. The above function denotes there is enough information to serve as the optimal feature set for the identification 0task when the cumulative contribution value of singular vectors from the first one to the λ-th one reaches a value, namely, the threshold *T*′. Thus, through the threshold *T*′, only a part of features were selected and then formed an optimal feature set, which made the model simple and fast to run.

### Machine Learning Methods

In order to distinguish between thermophilic and non-thermophilic proteins, SVM (Ding et al., 2016a, b; He et al., 2018; Qiao et al., 2018; Wei et al., 2018; Fu et al., 2019; Wang et al., 2019b), random forest [RF, (Ding et al., 2017; Wang et al., 2019a)], decision tree (Mohasseb et al., 2018; Li et al., 2019), and naïve Bayes [NB, (Rajaraman and Chokkalingam, 2014)] methods were used in our experiment. The first two methods were implemented and optimized in the python 3.7 environment with our edited code. All four methods were also tested in the Weka environment, yielding similar results.

### Evaluation of Performance

In order to evaluate the model performance, we used a 10-fold cross-validation scheme in our experiment and adopted three commonly used accuracy indicators for quantification (Jiang et al., 2013, 2018; Zeng et al., 2016; Wei et al., 2017a, b; Lu et al., 2018, 2019; Xiong et al., 2018; Chen et al., 2019; Ding et al., 2019; Lin et al., 2019; Shan et al., 2019; Shen et al., 2019; Xu et al., 2019; Yu and Gao, 2019; Yu et al., 2019a). The first indicator was sensitivity (Sn), which represents the ratio of the correctly identified thermophilic proteins and could be calculated as follows:

$$S\,n=\frac{T\,P}{T\,P+F\,N}\times 100\%,$$

where TP, TN, FP, and FN represent the number of the correctly identified thermophilic proteins, the number of the correctly indemnified non-thermophilic proteins, the number of non-thermophilic proteins predicted as thermophilic proteins, and the number of the thermophilic proteins predicted as non-thermophilic proteins, respectively (Lin and Chen, 2011).

The second indicator was specificity (Sp), which denotes the percentage of the correctly identified non-thermophilic proteins among all non-thermophilic observations. Sp was defined as follows:

$$S\,p=\frac{T\,N}{T\,N+F\,P}\times 100\%.$$

The last indicator was accuracy (ACC), which reflected the percentage of correctly recognized thermophilic and non-thermophilic proteins among all observations, written as follows:

$$A\,C\,C=\frac{T\,N+T\,P}{T\,N+F\,P+F\,N+F\,P}\times 100\%.$$

## Results

**FIGURE 2:**
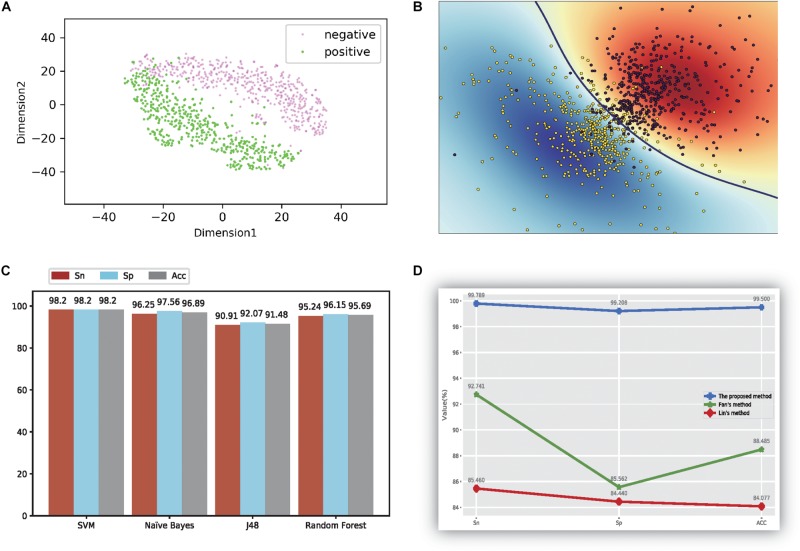
The figure of model performance. **(A)** The first two dimensions of the result of compression characteristics of the TSNE method; **(B)** the figure of the ultra-classification surface of SVM method; **(C)** The accuracy values of four different models; **(D)** the comparison results with other methods.

Our experiments were performed on the basis of qualitative evaluation, quantitative analysis, and comparison with other counterparts, as shown in Figure 2. The data were calculated using 500 randomly selected thermophilic proteins and 500 randomly selected non-thermophilic proteins, and experiments were evaluated in 10-fold cross-validation format.

First, we evaluated the proposed method using qualitative analysis. In this analysis, all feature data were reduced to 12 dimensions through the PCA method. Furthermore, the t-SNE method (van der Maaten and Hinton, 2012; van der Maaten, 2014) is one of the powerful visualization tools for showing the structure of high-dimension data. Thus, we used the t-SNE method (van der Maaten and Hinton, 2012; van der Maaten, 2014) to differentiate thermophilic and non-thermophilic proteins in the figure. Additionally, the t-SNE method used here was not a part of the proposed model, but was a display tool of the experiment data. The first two features of the results using the t-SNE method are plotted in Figure 2A; from these data, a distinct boundary was observed for separating thermophilic and non-thermophilic observations. Moreover, it was easy to distinguish thermophilic proteins from non-thermophilic proteins.

In order to verify these findings, SVM was used to train and test the 12-dimensional data, and the results are shown in Figure 2B. Both types of proteins were separated successfully using this method. This phenomenon directly demonstrated that our proposed data had good separation quality and the SVM method had strong recognition ability for thermophilic proteins and non-thermophilic data.

Second, the processed data were tested using the other three machine learning methods, as detailed in Figure 2C. For every method, we also calculated three accuracy indicators: Sn, Sp, and ACC. The results showed that the SVM yielded the highest values for all three indicators, and all values reached at least 98.2%. NB also showed higher accuracy, with values of 96.25%, 97.56%, and 96.89%, respectively. The accuracy of the random forest model was higher than that of J48, for which the average value was only 91.48%.

Our method was also compared with the results of Lin (Lin and Chen, 2011) and the method of using the same dataset (Fan et al., 2016). The results are shown in Figure 2D. Notably, our method got the highest accuracy values based on the results of the MRMD methods, which denotes our proposed method outperformed the method described by Lin (Lin and Chen, 2011). Additionally, the performance of the proposed method was better than the effects described by Fan et al. (2016) too, suggesting that the proposed method could be a state-of-the-art model in current research.

**FIGURE 3:**
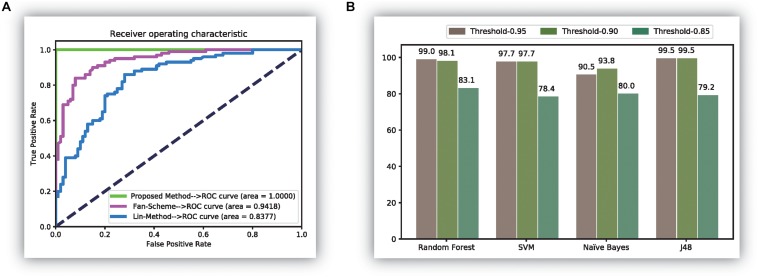
The comparison results of experiments. **(A)** The receiver operation characteristic (ROC) curve of three methods; **(B)** the results of experiments over the database (Fan et al., 2016).

Features using the original dipeptides were also tested in our study. All reduced features in our feature set were replaced with the original dipeptides. From the accuracy data shown in Figure 2D, the ability to distinguish thermophilic proteins from non-thermophilic ones was lower than that using the reduced amino acid dipeptides. Additionally, the receiver operating characteristic (ROC) curve was also plotted, which could be seen in Figure 3A. It is easy to found that the results of the ROC curve verified the identification efficiency of the proposed method too.

Finally, the newly released thermophilic protein database (Mohasseb et al., 2018) is also tested through the proposed method in this manuscript. In the experiment, we selected 106 thermophilic proteins and 101 psychrophilic proteins from the database. All those data can be downloaded on the website: http://www.labio.info/index-1therm.html. In the experiment, we did three experiments using three different thresholds in the correlation analysis step. The experiments are given in Figure 3B, from which it was easy to find that the identification accuracy was bigger than 0.97 in most cases when using the threshold of 0.95 and 0.90. It also showed that the classification efficiency was not ideal when using the threshold 0.85. The reason for this phenomenon may be the calculated features of the current data have a stronger correlation between each other than the previous thermophilic protein database. Thus, in this condition, a big value than 0.85 is needed to identify the thermophilic proteins accurately. It is worth noting that the results in this figure verified the perfect identification ability of the proposed method.

## Discussion

**FIGURE 4:**
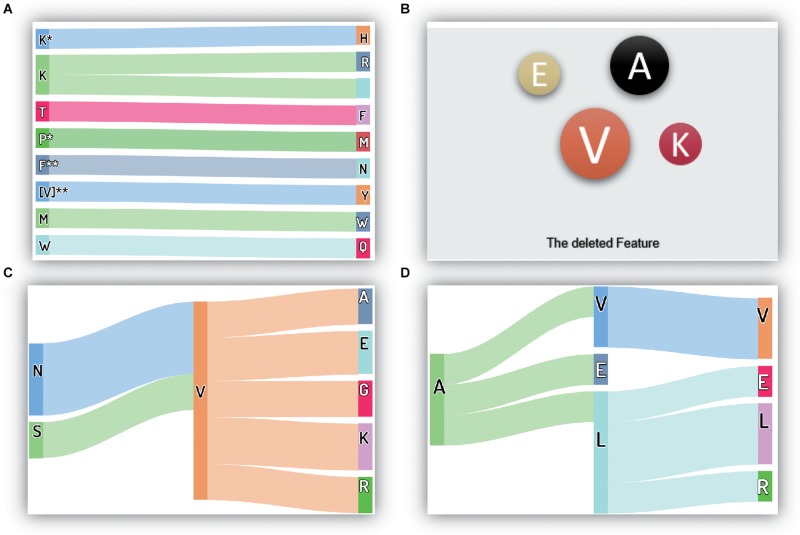
The critical and removed features in the proposed method: **(A)** the most important features; **(B)** the deleted amino acid frequency features; **(C)** the deleted reduced-depiptides **(I)**; **(D)** the deleted reduced-depiptides **(II)**. The symbol “*” means any one of the 20 amino acids, it may be “A”, “C”, “P”, or others. Besides, “**” has the same meaning; it represents a two-letter combination of 20 amino acids, “AA”, “DC”, “VP”, for example.

Many features are removed from the original feature set during correlation analysis and MRMD feature selection. Moreover, these removed features are typically not crucial or redundant for performing thermophilic protein recognition. However, the selection of features to remove and retain is essential, and further studies are needed to evaluate such approaches. Thus, in this study, we evaluated the removed features, as depicted in Figure 4.

The 10 most critical original features are shown in Figure 4A, and under our proposed model framework, the feature values of K^∗^H, KR, TF, P^∗^M, F^∗∗^N, I^∗∗^Y/V^∗∗^Y, MW, and WQ (where ^∗^ represents a gap in the residues) showed significant contributions to the recognition of thermophilic proteins. Additionally, residue K also plays a vital role in enhancing thermostability. Interestingly, our conclusions regarding residue K were consistent with the results of Lin (Lin and Chen, 2011).

For the removed features, the results are shown in Figures 4B–D. There were four types of components in the final feature set: ACC features, physicochemical characteristics, amino acid frequencies (the first 20 features in the 188D feature), and reduced amino acid dipeptides. Approximately half of the physicochemical characteristics were deleted from the original feature set, and there were only a few reserved physicochemical characteristics in the first 50 crucial features. Thus, we concluded that the physicochemical characteristics were essential features, but not the most essential features, for this recognition task. Accordingly, we did not analyze the details of the removed physicochemical characteristics. We also showed that only three ACC features were excluded from the final feature set, and the remaining 15 ACC features were retained, reflecting the crucial roles of the ACC features in this recognition task.

The amino acid frequency, which was one of the first 20 features in the 188D feature set, included only four residues removed from the feature set. These four residues were V (Ile and Val), A, E, and K, which had little contribution to recognizing thermophilic protein and non-thermophilic proteins. Interestingly, the reduced amino acid V, which included both Ile and Val, was also deleted. It is worth noting that the amino acid V appeared later in this manuscript denotes the reduced V, namely, both Ile and Val. This finding indicated that both Ile and Val were redundant and did not contribute to the identification task. If we used the original amino acid dipeptide features, additional useless features, including IA, I^∗^A, and I^∗∗^A, etc., would also be observed in the feature set. The number of additional redundant features in the original dipeptides could be as high as 39 if compared with the reduced amino acid dipeptides. As shown in Figure 2D, the smallest prediction accuracy was obtained, and represented those many additional useless features caused the classification model fail in the overfitting state when using the original dipeptides. Additionally, this observation could explain why the accuracy increased significantly when using the reduced amino acid dipeptides.

There were three types of dipeptides, expressed as AA (λ = 0), A^∗^A (λ = 1), and A^∗∗^A (λ = 2). The numbers of these types of removed dipeptides were 60, 61, and 71, respectively. To conveniently visualize these data, we counted the numbers of the same dipeptide (omitting the symbols ^∗^ and ^∗∗^). If a dipeptide appeared more than twice, it was drawn in the figure. Thus, if the dipeptide NV was shown in the figure, there were at least two types of dipeptides, i.e., NV, N^∗^V, or N^∗∗^V, in the removed feature set.

All discovered dipeptides were classified into two parts, as shown in Figures 4C,D. The reduced dipeptides in Figure 4C were dipeptides having relationships with the reduced residue V, verifying the reduced power of the recognition task in the above analysis. Moreover, residue V enabled the discovery of seven related dipeptides in the removed features. This phenomenon demonstrated that residue V and some dipeptides containing V were insensitive to the recognition task under our proposed model framework. Figure 4D also shows another seven removed dipeptides, including VV, AV, AE, AL, LE, LL, and LR.

These results provide insights into the design of stable mutants to increase protein thermostability.

## Conclusion

In this study, we aimed to develop an approach to distinguish thermophilic proteins from non-thermophilic proteins; to this end, a recognition method that combined mixed features of proteins and a machine learning method was established. First, an amino acid reduction method was introduced to reduce the categories of amino acids. Nest, we calculated the physicochemical characteristics, ACC, and reduced dipeptides of thermophilic and non-thermophilic proteins. After performing a dimension reduction step using correlation analysis, the MRMD method, and PCA, an optimal feature set was obtained. Finally, machine learning methods were used to train and predict feature data, and the results revealed that the proposed model could identify 98.2% of thermophilic proteins and non-thermophilic proteins if the data were operated in a 10-fold cross-validation mode. Furthermore, the feature values of K^∗^H, KR, TF, P^∗^M, F^∗∗^N, V^∗∗^Y, MW, and WQ were found to play vital roles in thermostability, and some residues and dipeptides, including V (Ile and Val), A, E, K, NV, VG, VA, AE, AL, and LE, were not important for identifying thermostability. As discussed in previous studies (Liu and Li, 2019; Liu and Zhu, 2019), the web-server is very important. In our future work, our research will focus on developing a free webserver that could provide a platform to test the currently proposed method using an easily accessible approach.

## Data Availability Statement

The datasets generated for this study can be found in the http://www.labio.info/index-1therm.html.

## Author Contributions

CF and YL: conceptualization. CF: methodology, software, and writing – original draft preparation. YL, JZ, and XL: validation. DY: formal analysis. ZM and XL: investigation. ZM: resources. XL: data curation. DY, YL, and JZ: writing – review and editing, supervision. CF and XL: visualization. DY and ZM: project administration. YL and JZ: funding acquisition. All authors have read and agreed to the published version of the manuscript.

## Conflict of Interest

The authors declare that the research was conducted in the absence of any commercial or financial relationships that could be construed as a potential conflict of interest.

## Footnotes

1 http://lin-group.cn/server/Lypred/

## References

[B1] Bhola A. Singh S. (2018). Gene selection using high dimensional gene expression data: an appraisal. *Curr. Bioinf.* 13 225–233.

[B2] Bleicher L. Prates E. T. Gomes T. C. F. Silveira R. L. Nascimento A. S. Rojas A. L. (2011). Molecular basis of the thermostability and thermophilicity of laminarinases: x-ray structure of the hyperthermostable laminarinase from rhodothermus marinus and molecular dynamics simulations. *J. Phys. Chem. B* 115 7940–7949. 10.1021/jp200330z 21619042

[B3] Cai C. Z. Han L. Y. Ji Z. L. Chen X. Chen Y. Z. (2003). SVM-Prot: web-based support vector machine software for functional classification of a protein from its primary sequence. *Nucleic Acids Res.* 31 3692–3697. 12824396 10.1093/nar/gkg600 PMC169006

[B4] Chen C. Zhang Q. M. Ma Q. Yu B. (2019). LightGBM-PPI: Predicting protein-protein interactions through LightGBM with multi-information fusion. *Chemometr. Intell. Labor. Syst.* 191 54–64.

[B5] Chen X. X. Tang H. Li W. C. Wu H. Chen W. Ding H. (2016). Identification of bacterial cell wall lyases via pseudo amino acid composition. *Biomed. Res. Int.* 2018:8. 10.1155/2016/1654623 PMC4942628 27437396

[B6] Cheng L. Wang P. P. Tian R. Wang S. Guo Q. H. Luo M. (2019). LncRNA2Target v2.0: a comprehensive database for target genes of lncRNAs in human and mouse. *Nucleic Acids Res.* 47 D140–D144. 30380072 10.1093/nar/gky1051 PMC6323902

[B7] Das R. Gerstein M. (2000). The stability of thermophilic proteins: a study based on comprehensive genome comparison. *Funct. Integr. Genom.* 1 76–88. 11793224 10.1007/s101420000003

[B8] Ding Y. J. Tang J. J. Guo F. (2016a). Identification of protein-protein interactions via a novel matrix-based sequence representation model with amino acid contact information. *Int. J. Mol. Sci.* 17:14. 10.3390/ijms17101623 27669239 PMC5085656

[B9] Ding Y. J. Tang J. J. Guo F. (2016b). Predicting protein-protein interactions via multivariate mutual information of protein sequences. *BMC Bioinf.* 17:13. 10.1186/s12859-016-1253-9 27677692 PMC5039908

[B10] Ding Y. J. Tang J. J. Guo F. (2017). Identification of drug-target interactions via multiple information integration. *Inform. Sci.* 418 546–560. 15840709

[B11] Ding Y. J. Tang J. J. Guo F. (2019). Identification of drug-side effect association via multiple information integration with centered kernel alignment. *Neurocomputing* 325 211–224.

[B12] Ding Y. R. Cai Y. J. Zhang G. X. Xu W. B. (2004). The influence of dipeptide composition on protein thermostability. *FEBS Lett.* 569 284–288. 15225649 10.1016/j.febslet.2004.06.009

[B13] Dong Q. W. Zhou S. G. Guan J. H. (2009). A new taxonomy-based protein fold recognition approach based on autocross-covariance transformation. *Bioinformatics* 25 2655–2662. 10.1093/bioinformatics/btp500 19706744

[B14] Fan G. L. Liu Y. L. Wang H. (2016). Identification of thermophilic proteins by incorporating evolutionary and acid dissociation information into Chou’s general pseudo amino acid composition. *J. Theor. Biol.* 407 138–142. 10.1016/j.jtbi.2016.07.010 27396359

[B15] Fu X. Z. Ke L. X. Cai L. J. Chen X. T. Ren X. B. Gao M. Y. (2019). Improved prediction of cell-penetrating peptides via effective orchestrating amino acid composition feature representation. *IEEE Access.* 7 163547–163555.

[B16] Fu X. Z. Zhu W. Liao B. Cai L. J. Peng L. H. Yang J. L. (2018). Improved DNA-binding protein identification by incorporating evolutionary information into the chou’s PseAAC. *IEEE Access.* 6 66545–66556.

[B17] Fukuchi S. Nishikawa K. (2001). Protein surface amino acid compositions distinctively differ between thermophilic and mesophilic bacteria. *J. Mol. Biol.* 309 835–843. 11399062 10.1006/jmbi.2001.4718

[B18] Gromiha M. M. (2001). Important inter-residue contacts for enhancing the thermal stability of thermophilic proteins. *Biophys. Chem.* 91 71–77. 11403885 10.1016/s0301-4622(01)00154-5

[B19] Gromiha M. M. Oobatake M. Sarai A. (1999). Important amino acid properties for enhanced thermostability from mesophilic to thermophilic proteins. *Biophys. Chem.* 82 51–67. 10584295 10.1016/s0301-4622(99)00103-9

[B20] Gromiha M. M. Pathak M. C. Saraboji K. Ortlund E. A. Gaucher E. A. (2013). Hydrophobic environment is a key factor for the stability of thermophilic proteins. *Proteins-Struct. Funct. Bioinf.* 81 715–721. 10.1002/prot.24232 23319168

[B21] Guo J. N. Luk L. Y. P. Loveridge E. J. Allemann R. K. (2014). Thermal adaptation of dihydrofolate reductase from the moderate thermophile *Geobacillus stearothermophilus*. *Biochemistry* 53 2855–2863. 10.1021/bi500238q 24730604 PMC4065160

[B22] Guo Y. Z. Yu L. Z. Wen Z. N. Li M. L. (2008). Using support vector machine combined with auto covariance to predict proteinprotein interactions from protein sequences. *Nucleic Acids Res.* 36 3025–3030. 18390576 10.1093/nar/gkn159 PMC2396404

[B23] He J. J. Fang T. Zhang Z. Z. Huang B. Zhu X. L. Xiong Y. (2018). PseUI: pseudouridine sites identification based on RNA sequence information. *BMC Bioinf.* 19:11. 10.1186/s12859-018-2321-0 30157750 PMC6114832

[B24] Hua J. P. Tembe W. D. Dougherty E. R. (2009). Performance of feature-selection methods in the classification of high-dimension data. *Pattern Recogn.* 42 409–424.

[B25] Jiang L. M. Xiao Y. K. Ding Y. J. Tang J. J. Guo F. (2018). FKL-Spa-LapRLS: an accurate method for identifying human microRNA-disease association. *BMC Genomics* 19:15. 10.1186/s12864-018-5273-x 30598109 PMC6311941

[B26] Jiang Q. H. Wang G. H. Jin S. L. Li Y. Wang Y. D. (2013). Predicting human microRNA-disease associations based on support vector machine. *Int. J. Data Min. Bioinf.* 8 282–293. 24417022 10.1504/ijdmb.2013.056078

[B27] Jin J. Miao Y. Y. Daly I. Zuo C. L. Hu D. W. Cichocki A. (2019). Correlation-based channel selection and regularized feature optimization for MI-based BCI. *Neural Netw.* 118 262–270. 10.1016/j.neunet.2019.07.008 31326660

[B28] Li M. J. Xu H. H. Deng Y. (2019). Evidential decision tree based on belief entropy. *Entropy* 21 14.

[B29] Li Y. Q. Fang J. W. (2010). Distance-dependent statistical potentials for discriminating thermophilic and mesophilic proteins. *Biochem. Biophys. Res. Commun.* 396 736–741. 10.1016/j.bbrc.2010.05.005 20451495 PMC2891751

[B30] Lin H. Chen W. (2011). Prediction of thermophilic proteins using feature selection technique. *J. Microbiol. Methods* 84 67–70. 10.1016/j.mimet.2010.10.013 21044646

[B31] Lin X. Quan Z. Wang Z.-J. Huang H. Zeng X. (2019). A novel molecular representation with BiGRU neural networks for learning atom. *Brief. Bioinf.* 10.1093/bib/bbz125 31729524

[B32] Liu B. (2019). BioSeq-Analysis: a platform for DNA, RNA and protein sequence analysis based on machine learning approaches. *Brief. Bioinform.* 20 1280–1294. 10.1093/bib/bbx165 29272359

[B33] Liu B. Li K. (2019). iPromoter-2L2.0: identifying promoters and their types by combining smoothing cutting window algorithm and sequence-based features. *Mol. Ther. Nucleic Acids* 18 80–87. 10.1016/j.omtn.2019.08.008 31536883 PMC6796744

[B34] Liu B. Liu F. L. Wang X. L. Chen J. J. Fang L. Y. Chou K. C. (2015). Pse-in-one: a web server for generating various modes of pseudo components of DNA, RNA, and protein sequences. *Nucleic Acids Res.* 43 W65–W71. 25958395 10.1093/nar/gkv458 PMC4489303

[B35] Liu B. Wang S. Y. Dong Q. W. Li S. M. Liu X. (2016). Identification of DNA-binding proteins by combining auto-cross covariance transformation and ensemble learning. *IEEE Trans. Nanobiosci.* 15 328–334. 10.1109/TNB.2016.2555951 28113908

[B36] Liu B. Zhu Y. L. (2019). ProtDec-LTR3.0: protein remote homology detection by incorporating profile-based features into learning to rank. *IEEE Access.* 7 102499–102507.

[B37] Liu X. L. Lu J. L. Hu X. H. (2011). Predicting thermophilic proteins with pseudo amino acid composition: approached from chaos game representation and principal component analysis. *Protein Peptide Lett.* 18 1244–1250. 21787282 10.2174/092986611797642661

[B38] Liu Y. M. Wang X. L. Liu B. (2019). A comprehensive review and comparison of existing computational methods for intrinsically disordered protein and region prediction. *Brief. Bioinform.* 20 330–346. 10.1093/bib/bbx126 30657889

[B39] Lu X. G. Li X. Liu P. Qian X. Miao Q. M. Peng S. L. (2018). The integrative method based on the module-network for identifying driver genes in cancer subtypes. *Molecules* 23:15. 10.3390/molecules23020183 29364829 PMC6099653

[B40] Lu X. G. Qian X. Li X. Miao Q. M. Peng S. L. (2019). DMCM: a data-adaptive mutation clustering method to identify cancer-related mutation clusters. *Bioinformatics* 35 389–397. 10.1093/bioinformatics/bty624 30010784

[B41] Meruelo A. D. Han S. K. Kim S. Bowie J. U. (2012). Structural differences between thermophilic and mesophilic membrane proteins. *Protein Sci.* 21 1746–1753. 23001966 10.1002/pro.2157 PMC3527711

[B42] Modarres H. P. Mofrad M. R. Sanati-Nezhad A. (2018). ProtDataTherm: a database for thermostability analysis and engineering of proteins. *PLoS ONE* 13:9. 10.1371/journal.pone.0191222 29377907 PMC5788348

[B43] Mohasseb A. Bader-El-Den M. Cocea M. (2018). Question categorization and classification using grammar based approach. *Inform. Process. Manage.* 54 1228–1243.

[B44] Mwangi B. Tian T. S. Soares J. C. (2014). A review of feature reduction techniques in neuroimaging. *Neuroinformatics* 12 229–244. 10.1007/s12021-013-9204-3 24013948 PMC4040248

[B45] Nakariyakul S. Liu Z. P. Chen L. N. (2012). Detecting thermophilic proteins through selecting amino acid and dipeptide composition features. *Amino Acids* 42 1947–1953. 10.1007/s00726-011-0923-1 21547362

[B46] Price A. L. Patterson N. J. Plenge R. M. Weinblatt M. E. Shadick N. A. Reich D. (2006). Principal components analysis corrects for stratification in genome-wide association studies. *Nat. Genet.* 38 904–909. 16862161 10.1038/ng1847

[B47] Qiao Y. H. Xiong Y. Gao H. Y. Zhu X. L. Chen P. (2018). Protein-protein interface hot spots prediction based on a hybrid feature selection strategy. *BMC Bioinf.* 19:16. 10.1186/s12859-018-2009-5 29334889 PMC5769548

[B48] Qu K. Y. Wei L. Y. Yu J. T. Wang C. Y. (2019). Identifying plant pentatricopeptide repeat coding gene/protein using mixed feature extraction methods. *Front. Plant Sci.* 9:10. 10.3389/fpls.2018.01961 30687359 PMC6335366

[B49] Rajaraman S. Chokkalingam A. (2014). Classification of denver system of chromosomes using similarity classifier guided by OWA operators. *Curr. Bioinf.* 9 499–508.

[B50] Saraboji K. Gromiha M. M. Ponnuswamy M. N. (2005). Importance of main-chain hydrophobic free energy to the stability of thermophilic proteins. *Int. J. Biol. Macromol.* 35 211–220. 15811476 10.1016/j.ijbiomac.2005.02.003

[B51] Shan X. Q. Wang X. G. Li C. D. Chu Y. Y. Zhang Y. F. Xiong Y. (2019). Prediction of CYP450 enzyme-substrate selectivity based on the network-based label space division method. *J. Chem. Inform. Model.* 59 4577–4586. 10.1021/acs.jcim.9b00749 31603319

[B52] Shen Y. N. Tang J. J. Guo F. (2019). Identification of protein subcellular localization via integrating evolutionary and physicochemical information into Chou’s general PseAAC. *J. Theor. Biol.* 462 230–239. 10.1016/j.jtbi.2018.11.012 30452958

[B53] Song L. Li D. P. Zeng X. X. Wu Y. F. Guo L. Zou Q. (2014). nDNA-prot: identification of DNA-binding proteins based on unbalanced classification. *BMC Bioinform.* 15:10. 10.1186/1471-2105-15-298 25196432 PMC4165999

[B54] Susko E. Roger A. J. (2007). On reduced amino acid alphabets for phylogenetic inference. *Mol. Biol. Evol.* 24 2139–2150. 17652333 10.1093/molbev/msm144

[B55] Takai K. Nakamura K. Toki T. Tsunogai U. Miyazaki M. Miyazaki J. (2008). Cell proliferation at 122 degrees C and isotopically heavy CH4 production by a hyperthermophilic methanogen under high-pressure cultivation. *Proc. Natl. Acad. Sci. U.S.A.* 105 10949–10954. 10.1073/pnas.0712334105 18664583 PMC2490668

[B56] Tang H. Cao R. Z. Wang W. Liu T. S. Wang L. M. He C. M. (2017). A two-step discriminated method to identify thermophilic proteins. *Int. J. Biomathemat.* 10:8.

[B57] Thibeault C. M. Srinivasa N. (2013). Using a hybrid neuron in physiologically inspired models of the basal ganglia. *Front. Comput. Neurosci.* 7:17. 10.3389/fncom.2013.00088 23847524 PMC3701869

[B58] van der Maaten L. (2014). Accelerating t-SNE using Tree-Based Algorithms. *J. Mach. Learn. Res.* 15 3221–3245.

[B59] van der Maaten L. Hinton G. (2012). Visualizing non-metric similarities in multiple maps. *Mach. Learn.* 87 33–55. 10.1186/s12859-018-2537-z 30577738 PMC6302369

[B60] Vieille C. Zeikus G. J. (2001). Hyperthermophilic enzymes: sources, uses, and molecular mechanisms for thermostability. *Microbiol. Mol. Biol. Rev.* 65 1–43. 11238984 10.1128/MMBR.65.1.1-43.2001 PMC99017

[B61] Wang D. Yang L. Fu Z. Q. Xia J. B. (2011). Prediction of thermophilic protein with pseudo amino acid composition: an approach from combined feature selection and reduction. *Protein Peptide Lett.* 18 684–689. 21413920 10.2174/092986611795446085

[B62] Wang G. H. Luo X. M. Wang J. N. Wan J. Xia S. L. Zhu H. (2018). MeDReaders: a database for transcription factors that bind to methylated DNA. *Nucleic Acids Res.* 46 D146–D151. 29145608 10.1093/nar/gkx1096 PMC5753207

[B63] Wang X. Y. Yu B. Ma A. J. Chen C. Liu B. Q. Ma Q. (2019a). Protein-protein interaction sites prediction by ensemble random forests with synthetic minority oversampling technique. *Bioinformatics* 35 2395–2402. 10.1093/bioinformatics/bty995 30520961 PMC6612859

[B64] Wang Y. Shi F. Q. Cao L. Y. Dey N. Wu Q. Ashour A. S. (2019b). Morphological segmentation analysis and texture-based support vector machines classification on mice liver fibrosis microscopic images. *Curr. Bioinf.* 14 282–294.

[B65] Wei L. Y. Wan S. X. Guo J. S. Wong K. K. L. (2017a). A novel hierarchical selective ensemble classifier with bioinformatics application. *Artif. Intell. Med.* 83 82–90. 10.1016/j.artmed.2017.02.005 28245947

[B66] Wei L. Y. Xing P. W. Zeng J. C. Chen J. X. Su R. Guo F. (2017b). Improved prediction of protein-protein interactions using novel negative samples, features, and an ensemble classifier. *Artif. Intell. Med.* 83 67–74. 10.1016/j.artmed.2017.03.001 28320624

[B67] Wei L. Y. Zhou C. Chen H. R. Song J. N. Su R. (2018). ACPred-FL: a sequence-based predictor using effective feature representation to improve the prediction of anti-cancer peptides. *Bioinformatics* 34 4007–4016. 10.1093/bioinformatics/bty451 29868903 PMC6247924

[B68] Wu L. C. Lee J. X. Huang H. D. Liu B. J. Horng J. T. (2009). An expert system to predict protein thermostability using decision tree. *Exp. Syst. Appl.* 36 9007–9014.

[B69] Xiao J. Liu S. D. Hu L. Wang Y. (2018). Filtering method of rock points based on BP neural network and principal component analysis. *Front. Comput. Sci.* 12:1149–1159. 10.1007/s11704-016-6170-6

[B70] Xiong Y. Wang Q. K. Yang J. C. Zhu X. L. Weil D. Q. (2018). PredT4SE-Stack: prediction of bacterial type IV secreted effectors from protein sequences using a stacked ensemble method. *Front. Microbiol.* 9:9. 10.3389/fmicb.2018.02571 PMC6212463 30416498

[B71] Xu H. Zeng W. H. Zhang D. F. Zeng X. X. (2019). MOEA/HD: a multiobjective evolutionary algorithm based on hierarchical decomposition. *IEEE Trans. Cybernet.* 49 517–526. 10.1109/TCYB.2017.2779450 29990272

[B72] Xu L. Liang G. M. Shi S. H. Liao C. R. (2018). SeqSVM: a sequence-based support vector machine method for identifying antioxidant proteins. *Int. J. Mol. Sci.* 19:11. 10.3390/ijms19061773 29914044 PMC6032279

[B73] Xu R. F. Zhou J. Y. Liu B. Yao L. He Y. L. Zou Q. (2014). enDNA-Prot: identification of DNA-binding proteins by applying ensemble learning. *Biomed. Res. Int.* 2014:10. 10.1155/2014/294279 PMC4058174 24977146

[B74] Yang W. Zhu X. J. Huang J. Ding H. Lin H. (2019). A brief survey of machine learning methods in protein sub-golgi localization. *Curr. Bioinf.* 14 234–240.

[B75] Yu B. Qiu W. Chen C. Ma A. Jiang J. Zhou H. (2019a). SubMito-XGBoost: predicting protein submitochondrial localization by fusing multiple feature information and eXtreme gradient boosting. *Bioinformatics* 36, 1074–1081. 10.1093/bioinformatics/btz734 31603468

[B76] Yu L. Yao S. Y. Gao L. Zha Y. H. (2019b). Conserved disease modules extracted from multilayer heterogeneous disease and gene networks for understanding disease mechanisms and predicting disease treatments. *Front. Genet.* 9:13. 10.3389/fgene.2018.00745 30713550 PMC6346701

[B77] Yu L. Gao L. (2019). Human pathway-based disease network. *IEEE-ACM Trans. Comput. Biol. Bioinf.* 16 1240–1249. 10.1109/TCBB.2017.2774802 29990107

[B78] Yu L. Su R. D. Wang B. B. Zhang L. Zou Y. P. Zhang J. (2017a). Prediction of novel drugs for hepatocellular carcinoma based on multi-source random walk. *IEEE-ACM Trans. Comput. Biol. Bioinf.* 14 966–977. 10.1109/TCBB.2016.2550453 27076463

[B79] Yu L. Zhao J. Gao L. (2017b). Drug repositioning based on triangularly balanced structure for tissue-specific diseases in incomplete interactome. *Artif. Intell. Med.* 77 53–63. 10.1016/j.artmed.2017.03.009 28545612

[B80] Zeng X. Lin Y. He Y. Lv L. Min X. Rodriguez-Paton A. (2019a). Deep collaborative filtering for prediction of disease genes. *IEEE-ACM Trans. Comput. Biol. Bioinf.* 10.1109/TCBB.2019.2907536 30932845

[B81] Zeng X. Zhong Y. Lin W. Zou Q. (2019b). Predicting disease-associated circular RNAs using deep forests combined with positive-unlabeled learning methods. *Brief. Bioinf.* 10.1093/bib/bbz080 31612203

[B82] Zeng X. X. Liao Y. L. Liu Y. S. Zou Q. (2017). Prediction and validation of disease genes using hetesim scores. *IEEE-ACM Trans. Comput. Biol. Bioinf.* 14 687–695. 10.1109/TCBB.2016.2520947 26890920

[B83] Zeng X. X. Zhang X. Zou Q. (2016). Integrative approaches for predicting microRNA function and prioritizing disease-related microRNA using biological interaction networks. *Brief. Bioinform.* 17 193–203. 10.1093/bib/bbv033 26059461

[B84] Zhang F. Ma A. J. Wang Z. Ma Q. Liu B. Q. Huang L. (2018). A central edge selection based overlapping community detection algorithm for the detection of overlapping structures in protein-protein interaction networks. *Molecules* 23:16. 10.3390/molecules23102633 30322177 PMC6222769

[B85] Zhang G. Y. Fang B. S. (2006a). Application of amino acid distribution along the sequence for discriminating mesophilic and thermophilic proteins. *Process Biochem.* 41 1792–1798.

[B86] Zhang G. Y. Fang B. S. (2006b). Discrimination of thermophilic and mesophilic proteins via pattern recognition methods. *Process Biochem.* 41 552–556.

[B87] Zhang G. Y. Fang B. S. (2007). LogitBoost classifier for discriminating thermophilic and mesophilic proteins. *J. Biotechnol.* 127 417–424. 17045354 10.1016/j.jbiotec.2006.07.020

[B88] Zhang M. Li F. Y. Marquez-Lago T. T. Leier A. Fan C. Kwoh C. K. (2019). MULTiPly: a novel multi-layer predictor for discovering general, and specific types of promoters. *Bioinformatics* 35 2957–2965. 30649179 10.1093/bioinformatics/btz016 PMC6736106

[B89] Zhang X. Zou Q. Rodriguez-Paton A. Zeng X. X. (2019). Meta-path methods for prioritizing candidate disease miRNAs. *IEEE-ACM Trans. Comput. Biol. Bioinf.* 16 283–291. 10.1109/TCBB.2017.2776280 29990255

[B90] Zheng L. Huang S. Mu N. Zhang H. Zhang J. Chang Y. (2019). RAACBook: a web server of reduced amino acid alphabet for sequence-dependent inference by using Chou’s five-step rule. *Database* 2019, 1–12. 10.1093/database/baz131 PMC6893003 31802128

[B91] Zhou X. X. Wang Y. B. Pan Y. J. Li W. F. (2008). Differences in amino acids composition and coupling patterns between mesophilic and thermophilic proteins. *Amino Acids.* 34 25–33. 17710363 10.1007/s00726-007-0589-x

[B92] Zhu X. J. Feng C. Q. Lai H. Y. Chen W. Hao L. (2019). Predicting protein structural classes for low-similarity sequences by evaluating different features. *Knowl. Based Syst.* 163 787–793.

[B93] Zou Q. Chen L. Huang T. Zhang Z. G. Xu Y. G. (2017a). Machine learning and graph analytics in computational biomedicine. *Artif. Intell. Med.* 83 1–1. 28935226 10.1016/j.artmed.2017.09.003

[B94] Zou Q. Mrozek D. Ma Q. Xu Y. G. (2017b). Scalable data mining algorithms in computational biology and biomedicine. *Biomed. Res. Int.* 2017:3. 10.1155/2017/5652041 PMC5350399 28337450

[B95] Zou Q. Zeng J. C. Cao L. J. Ji R. R. (2016). A novel features ranking metric with application to scalable visual and bioinformatics data classification. *Neurocomputing* 173 346–354.

[B96] Zuo Y. C. Chen W. Fan G. L. Li Q. Z. (2013). A similarity distance of diversity measure for discriminating mesophilic and thermophilic proteins. *Amino Acids* 44 573–580. 10.1007/s00726-012-1374-z 22851052

